# Gut microbes, ageing & organ function: a chameleon in modern biology?

**DOI:** 10.15252/emmm.201809872

**Published:** 2019-08-14

**Authors:** Musarrat Maisha Reza, B Brett Finlay, Sven Pettersson

**Affiliations:** ^1^ Department of Neurobiology Care Sciences and Society (NVS) Karolinska Institutet Stockholm Sweden; ^2^ School of Biological Sciences Nanyang Technological University Singapore City Singapore; ^3^ Michael Smith Laboratories and the Departments of Biochemistry and Molecular Biology, and Microbiology and Immunology University of British Columbia Vancouver BC Canada; ^4^ Lee Kong Chian School of Medicine Nanyang Technological University Singapore City Singapore; ^5^ Singapore Centre for Environmental Life Sciences Engineering Nanyang Technological University Singapore City Singapore

**Keywords:** ageing, health, interventions, microbiome, philosophy, Ageing, Digestive System, Microbiology, Virology & Host Pathogen Interaction

## Abstract

All species, including humans, are cohabited by a myriad of microbial species, which massively influences body function in a diet‐, exercise‐ and age‐dependent manner. The microbiome composition differs between individuals, partly due to the polymorphic immune system, as well as the environment, making the microbe–host interplay unique in each one of us. Ageing is a gradual loss of function in part due to reduced repair mechanisms and accumulation of tissue damage through mechanisms largely unknown. Accumulating evidence suggests that our indigenous microbes, a known major regulator of human physiology, are also connected to regulate the ageing process through signalling pathways and metabolites though the biological mechanisms are unknown. At an ageing meeting in Singapore in 2018, investigators discussed the current understanding of microbe regulation and its impact on healthy ageing. This review summarizes the highlights from the meeting and conveys some of the new ideas that emerged around gut microbes and the biology of ageing. While highly speculative, an idea emerged in which gut microbes constantly respond and evolve to environmental cues, as part of an ageing process, thus serving as a second messenger to support and attenuate organ decline in a diet‐, gender‐ and age‐dependent manner.

**Glossary**
Bio‐active: – having a biological effect.
Calorific value: – energy contained in food, determined by measuring the heat produced by the complete breakdown of it.
Circadian rhythmicity: – is a roughly 24‐h cycle in the physiological processes of living beings. It is usually endogenously generated and can be modulated by external cues such as sunlight and temperature.
Cognitive ability: – general mental capability involving reasoning, problem‐solving, planning, abstract thinking, complex idea comprehension and learning from experience.
Cognitive function: – refers to multiple mental abilities such as learning, thinking, reasoning, attention, remembering, problem solving and decision making.
Degenerative pathways: – pathways resulting in changes that affect tissues and organs to deteriorate increasingly over time.
Disease susceptibility: – an increased likelihood of developing a disease based on the genetic make‐up, often due to the inheritance of a genetic variation from the parent.
Diurnal rhythmicity: – patterns of activity or behaviour that follow the day and night cycle, repeating every 24 h.
Dysbiosis: – a term used for a microbial imbalance on or inside the body, such as an impaired microbiota where microbiome become deranged, with predominant species being underrepresented and normally contained species increasing to fill the void.
Ecological niche: – the role and position a species has in its environment; how it meets its needs for food and shelter, how it survives, and how it reproduces.
Glycaemic index: – is the value assigned to foods based on how slowly or how quickly they cause increases in blood glucose levels. It is a number from 0 to 100 assigned to a food, with pure glucose arbitrarily given the value of 100.
Holistic interventions: – economical, non‐invasive, non‐pharmacologic alternatives to traditional medical care that expand the repertoire of therapies to manage and control diseases.
Induced pluripotent stem cell: – adult cells that have been genetically reprogrammed to an embryonic stem cell‐like state by being forced to express genes and factors important for maintaining the defining properties of embryonic stem cells.
Mass spectrometry: – an analytical technique that measures the mass‐to‐charge ratio of ions in order to quantify known materials, identify unknown compounds within a sample and to elucidate the structure and chemical properties of different molecules.
Metabolite: – intermediate end product of metabolism, usually a small molecules.
Metabolome map: – a map of the global collection of low molecular weight metabolites produced by cells during metabolism providing a functional readout of cellular activity and physiological status.
Metabolome: – complex set of small molecule chemicals found within a cell, organelle, tissue, biofluid or the entire organism.
Microbiome: – the microorganisms in a particular environment of the body or a part of the body.
Neural niche: – a zone in which stem cells are retained after embryonic development for the production of new cells for the nervous system.
Neurodegenerative diseases: – conditions that primarily affect the neurons in the brain and spinal cord causing them to die or be damaged. Examples of diseases would be Parkinson's, Alzheimer's and Huntington's.
Neurotoxic effects: – damage to the peripheral or central nervous systems due to the exposure to natural or man‐made toxic substances, which can alter the activity of the nervous system to disrupt or kill nerves.
Niche: – a specialized and specific zone or area.
Non‐senescent: – organisms that do not exhibit any measurable decline in survival characteristics such as strength or mobility with age, do not have a gradually increasing death rate with age and also do not exhibit any measurable reduction in reproductive ability with age.
Operant paradigm: – behaviour that is modifiable and controlled by its consequences.
Personalized nutrition: – an approach that uses information on individual characteristics to develop targeted nutritional advice, products, or services to assist individuals achieve lasting beneficial dietary behaviour changes.
Phenotype: – a composite of the individual's observable characteristics as a result of the interaction between its genotype with the environment.
Physiology: – the way in the body or specific body parts and organs function and interact with one another.
Plasticity: – the ability of living organisms to change their “state” in response to any stimuli and showing an adaptive response for optimal survival.
Polymorphic: – a discontinuous genetic variation resulting in the occurrence of different types of individuals among the members of a single species.
Post‐prandial: – during or relating to the period after meal: dinner or lunch.
Precision medicine: – also known otherwise as personalized medicine, is an approach to patient care that empowers healthcare providers to select and administer treatments most likely to be effective to patients based on the genetic understanding of their disease.
Symbiotic: – interaction between two different organisms living in close physical and long‐term biological association enabling a mutually benefiting relationship.

## Gut microbes and the host—a unique mosaic of microbes and organs that build and shape the human body

Throughout evolution, complex organisms have acquired prokaryotic organelles such as mitochondria and chloroplasts to optimize biological functions (Gilbert *et al*, 2012; Douglas, 2014). Human beings coexist in a complex symbiotic relationship with more than a 1,000 different bacterial species in and on our bodies (Qin *et al*, 2010). These indigenous microbes occupy multiple niches in a temporal and spatial fashion through mechanisms that are largely unknown. The predominant location of microbes is in the alimentary tract (Kundu *et al*, 2017), where they execute numerous functions including securing nutritional supply through highly sophisticated metabolic circuits.

Current models suggest that microbes massively influence host physiology (Brown *et al*, 2013; Hooper *et al*, 2001, 2002; Nicholson *et al*, 2012, Natarajan & Pluznick, 2014). The intensity and dynamics of these interactions between the host and the microbes are highly variable due to large differences in microbiome composition and diversity. Therefore, each individual is a unique composite of eukaryotic genes and microbes that collectively impact body function and well‐being in a diet‐, sex‐ and age‐dependent manner (Leser & Mølbak, 2009; Spor *et al*, 2011). That is, the mechanisms by which microbiome composition, richness, and metabolites tune organ maturation, formation of body homeostasis, and function in a human being in early life are likely to also be good predictors for microbiome‐associated regulation of both healthy ageing and diseases such as type II diabetes, cardiovascular perturbations, neurodegenerative diseases and cancer.

## Birth, postnatal life and the first acquisition of maternal microbes

The newborn mammalian offspring acquires colonizing microbes predominantly from the birth canal during vaginal delivery from the mother, thereby providing a trajectory of early maternal colonizers dominated by *Lactobacillus, Bifidobacter and Prevotella* spp (Dominguez‐Bello *et al*, 2010). This biologically selected pathway of child delivery is currently under threat due to the rapidly increasing number of children delivered by Caesarean section. Recent data suggest an early microbiome composition different to those undergoing vaginal delivery, thereby changing the evolutionary selected symbiotic vertical transmission of maternal microbes (Dunn *et al*, 2017).

In addition, highly controversial reports suggest that exposure to microbes may occur before birth in the womb (Bearfield *et al*, 2002; Markenson *et al*, 2003; Jiménez *et al*, 2005; Rautava *et al*, 2012; Hou *et al*, 2013; Stout *et al*, 2013; Aagaard *et al*, 2014; Mändar *et al*, 2015; Zheng *et al*, 2015; Verstraelen *et al*, 2016), thus challenging the sterile womb dogma (Stout *et al*, 2013). While fascinating, it remains to be excluded that the presence of these microbes may be experimental contamination processes. Less controversial are the reports showing that maternal microbes are also transmitted to newborns via breast milk (Heikkilä & Saris, 2003; Martín *et al*, 2003; Beasley & Saris, 2004), which provide them with approximately 8 × 10^4^ to 8 × 10^6^ bacteria daily (Heikkilä & Saris, 2003). That is, breastfeeding is a powerful way of enhancing the colonization process and increasing the microbial diversity and thus the maturation of the gut microbiome (higher levels of *Bifidobacterium* species, characterized by *Firmicutes* bacteria) (Stewart *et al*, 2018). As the growing child develops, the microbiome undergoes a continuum of changes in diversity and species. A longitudinal study was performed on stool samples from 903 children aged between 3 and 46 months as part of the Environmental Determinants of Diabetes in the Young (TEDDY) study. 16S rRNA gene sequencing and metagenomic sequencing revealed that the gut microbiome progresses in what the investigators characterize as a step‐dependent manner; an early developmental phase lasting up to 14 months of age, an expanding transitional phase up to 30 months of age followed by the entry into a stable phase up to 46 months (Stewart *et al*, 2018). These changes in early life coincide with the gradual exposure to different diets including solid food, which significantly impact and further diversify microbiome composition and diversity by a rapid expansion of anaerobes.

## Diet is a major driver of microbiome diversification

The young human gradually acquires the adult microbiome, resulting in what is commonly known as the mature microbiome (Borre *et al*, 2014). A trademark of the adult microbiome is its stability, which possesses the ability to mitigate transient effects of stress, a potential disruptor of microbial stability (Borre *et al*, 2014). Despite its stability, the gut microbes possess the ability to respond to environmental cues. Hence, this opens the door to therapeutic interventions specifically targeting the microbial community at the individual level. For example, differences in environmental factors (Kundu *et al*, 2017; Stewart *et al*, 2018) have been shown to influence microbiome composition and diversity.

A central function for gut microbes is their ability to digest incoming food material. Many of the homeostatic interactions between the mammalian host and its gut microbiome are indeed mutually beneficial. Gut microbes have the capacity to produce essential vitamins and amino acids (LeBlanc *et al*, 2011, 2013, 2017; Rossi *et al*, 2011) and convert host‐generated primary bile acids into bio‐active, secondary bile acids (David *et al*, 2014). In addition, microbes also metabolize dietary plant polysaccharides (fibre) (Fischbach & Sonnenburg, 2011) and support lipid absorption in the intestinal canal (Semova *et al*, 2012).

Another important area of future research is to identify key microbes that act like orchestral leaders, thereby guiding other microbes to support and maintain physiological functions. For example, microbes regulating tryptophan metabolism represent only 10% of the gut microbiome, suggesting a stringent control within the microbiome community at an individual level. Moreover, prolonged expression of indoles has been documented to support longevity in several animal models including flies, worms and mice (Sonowal *et al*, 2017). Interestingly, in a recent paper it was demonstrated that certain microbes, such as *Clostridium difficile,* possesses the ability to elicit signals to tryptophan metabolizing microbes through the accessory gene regulator 1 quorum‐sensing system (Atg1). Activation of Atg1 induces the machinery to secrete indoles by nearby microbe secretion (Kleino & Silverman, 2019). This mechanism is therefore a powerful virulence mechanism for *Clostridium difficile* to begin colonizing the alimentary tract, as indoles are known for bacterial replication. This example illustrates a situation where microbes and host exhibit opposite needs resulting in disease manifestation for the host. Future research should aim to identify, which microbes, among the commensal microbes possess this ability to regulate indole secretion. Are we losing those indole inducers as we age? Are there sexual dimorphisms in their numbers?

The gut microbiome also responds swiftly to dietary changes via microbial‐derived metabolites (De Filippo *et al*, 2010; Albenberg & Wu, 2014; Conlon & Bird, 2015) such as short‐chain fatty acids (SCFAs). SCFAs are known to regulate the mTOR pathway, one of the key longevity pathways (Cornu *et al*, 2013; Johnson *et al*, 2013; Passtoors *et al*, 2013; Henrique Mazucanti *et al*, 2015; Park *et al*, 2015). Interestingly, a gradual loss of SCFA by age has been reported, indicating a potential link between SCFA and ageing regulating pathways (Kundu *et al*, 2017). The glycaemic index is a system relying on measurement of blood sugar levels in response to different foods, while assuming that different individuals have identical glycaemic responses to food. This assumption was in fact challenged in the cited study, which suggests that different people have differential responses to identical foods (Zeevi *et al*, 2015), which confirms that there is no “one‐size‐fits‐all” diet that is optimal for everyone. Another interesting study recently reported that microbes producing SCFA possess the ability to increase expression of the hormone FGF21 (Pathak *et al*, 2018). The recent observation that FGF21 treatment using a gene therapy approach can combat diabetes (Jimenez *et al*, 2018) implies that food products can be used to increase microbe‐derived SCFA production, which may become a new intervention scheme to treat obesity and insulin resistance.

Venturing into personalized nutrition via individual personal profiling may produce a reduction in the post‐prandial glucose response and attenuate symptoms of pre‐ or type II diabetes (Franz *et al*, 2002; American Diabetes Association, 2008), since *what* we eat and *when* we eat impact our microbiome. This is an exciting and novel concept, given the extensive microbial differences between individuals and also an explorative avenue to attenuate accelerated ageing. Moreover, micronutrient intervention therapies will have much reduced side effects as compared to small molecule therapies.

Marine biologists have reported that microbes display diurnal rhythmicity. Further, like our own body clock, a large fraction of microbes are assumed to change functionality at different times of the day (Franz *et al*, 2002; American Diabetes Association, 2008). Host physiology and feeding rhythmicity determine microbiome clock function. Therefore, controlling feeding behaviour affects microbiome circadian rhythmicity and *vice versa* and represents another promising area of future intervention strategies (Franz *et al*, 2002; American Diabetes Association, 2008). These strategies may provide ways to tackle pandemic chronic diseases such as obesity and type II diabetes. It was estimated that in 2016 about 1.9 billion adults were overweight/obese (WHO, 2017a) and the number of people with diabetes reached 422 million in 2014 (WHO, 2017b). Given the reported association with changes in microbiome composition in obesity and diabetes (Ley *et al*, 2005; Tilg & Kaser, 2011; Devaraj *et al*, 2013) and that diet is the major regulator of microbiome composition (Hildebrandt *et al*, 2009; Moschen *et al*, 2012), it is worth exploring whether intervention strategies that change microbiome composition can attenuate disease burden.

Experiments conducted on ageing mice subjected to a 30% caloric restriction and fed with low fat diet that was enriched with *Lactobacillus* showed extended lifespan and reduced inflammation, as circulating lipopolysaccharides in the serum was reduced (Zhang *et al*, 2013), exemplifying the potential of diet manipulation in regulating the health of the elderly.

Large bowel microbiome function can be improved by employing various dietary strategies (Ziemer & Gibson, 1998; Laparra & Sanz, 2010). Compounds like acetate and butyrate that are SCFAs have large benefits in the gut. Acetylated starches are bound starches, which resist breakdown in the small intestine, facilitated by bacterial enzymes. An increase in resistant starch results in increased butyrate levels and consumption of foods like BARLEYmax, which has a low glycaemic index and significantly promotes bowel health (Aoe *et al*, 2018). Accumulating data suggest reduction in SCFA producing microbes as one ages (Biagi *et al*, 2013). While the mechanisms underlying this age‐dependent change are currently unknown, the BARLEYmax diet intervention holds promise for future design of synthetic diet interventions to increase the number of microbes producing SCFA among the elderly.

It is generally assumed that a diverse microbiome is strongly associated with a healthier body (Cho & Blaser, 2012), while the lack of diversity has been correlated with disease and mortality. Additionally, the diversity of our diet appears to proportionally affect our microbiome and enhances its adaptability to environmental perturbations (Bolnick *et al*, 2014; Heiman & Greenway, 2016).

Profiling of biofluids can help elucidate the function of the microbiome. Recently, an attractive diet for healthy ageing from the OmniHeart trial was shown to reduce blood pressure and triglycerides (Appel *et al*, 2005; Miller *et al*, 2006; Doménech *et al*, 2014). This diet contains several essential metabolites: guanidinoacetate and phenylacetylglutamine p‐cresol sulphate. The OmniHeart trial tested three different diets, all with the same calorific value. One diet was enriched in carbohydrates, the other in protein, and the third diet in unsaturated fat. The protein‐rich and unsaturated fat‐rich diets showed reduced blood pressure and blood lipids with a reduction in the risk of heart disease over a next 10‐year period (Appel *et al*, 2005; Miller *et al*, 2006; Doménech *et al*, 2014). While this diet is targeted specifically for the maintenance of a healthy heart, it also has significant beneficial effects on brain health and hence overall body health. Using the MIND diet, a hybrid of the Mediterranean and DASH diet suggests that it is possible to decrease the incidence of dementia and Alzheimer's disease (Tangney, 2014; Morris *et al*, 2015a,b). While further studies are highly warranted, it seems our grandparents’ wisdom can add value to modern day science, when they would tell us the benefits of eating a wide variety of food and consuming an inclusive diet. In fact, food intake that has limited diversity of animal and plant products (i.e. white flour and white sugar) severely restricts gut microbiome diversity (Heiman & Greenway, 2016). That is, exposing the body to either a plant‐ or animal‐based diet even temporally drastically changes the structure of the gut microbiome (David *et al*, 2014).

## Ageing and the microbiome

There is a rapid expansion of an ageing population worldwide. For example, 14% of the Australian population is over 65 and it is predicted to rise to 22% by 2061 (Australian Bureau of Statistics, 2013) and 28% of the population in Singapore will be aged 65 or older by 2030 (Siau, 2017). Hence, more resources should be allocated to address the role of gut microbes in regulating the biology of ageing in addition to other mechanistic studies revolving on the physiology and pathophysiology of ageing. Current data suggest that considerable changes in microbiome composition and diversity occur early in life when the expanding microbiome is to be formed and build the growing offspring (Kundu *et al*, 2017). Similarly, later in life, when body function declines, due to gradual loss of function and accumulation of organ damage, reduced number of stem cells and reduced ability to repair the ageing body, the microbiome appears to reduce in diversity and richness (O'Toole & Claesson, 2010). Interestingly, the reduction of diversity and richness are associated with the degree of social interactions with other humans. Reduced interactions with other people result in reduced microbial diversity, thus implying social interactions as another way to support microbiome richness and diversity (O'Toole & Claesson, 2010). Because reduced diversity has been associated with increased susceptibility to disease acquisition, further experiments are highly warranted especially in the light of the rapidly growing population of elderly often associated with increased isolation and detachment from society (Cho & Blaser, 2012).

It has been shown in one of the initial works on microbiome and ageing using sequencing of the 16S rDNA from elderly Caucasians over 65 years of age that a significant shift occurs in the microbial phyla during ageing, from *Firmicutes* to *Bacteroidetes* (Claesson *et al*, 2011). While currently unknown, we speculate that these changes in microbiome composition of an elderly person are an internal image of the gradual accumulation of reduced organ function and reduced ability to maintain barrier integrity (Kundu *et al*, 2017). Interestingly, a study demonstrated that a gradual loss of microbiome diversity is associated with a loss of social communication where the most reduced microbiome diversity was observed in individuals with reduced social interactions (Cho & Blaser, 2012; Kundu *et al*, 2017). Like humans, microbial communities work in concerted action to achieve common goals, like reducing energy consumption to access nutrients, etc. Hence, a microbiome with reduced diversity may therefore have multi‐detrimental consequences for the ageing human being.

**Figure 1:**
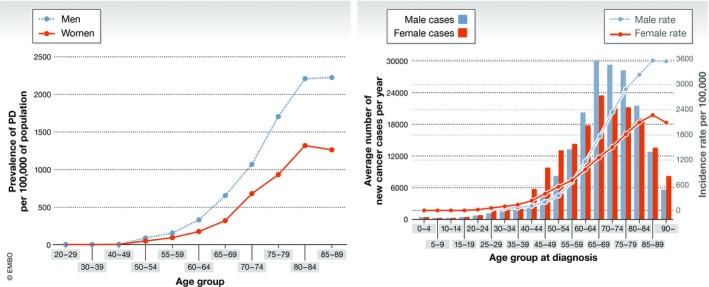
Disease prevalence in men and women with increasing age (Left) Graph showing the prevalence rates (per 100,000) of Parkinson's in UK by age and gender in the year 2015. Graph (primary data) has been redrawn with the appropriate authorization (Parkinson's, UK 2018). The incidence and prevalence of Parkinson's in the UK (*London, UK*). (Right) Graph showing the average number of new cases of cancer per year for each age bracket as well as the incidence rate of cancer per 100,000 cases in the UK from 2013 to 2015. Graph (primary data) has been redrawn with the appropriate authorization (Cancer Research UK 2018). Cancer incidence by age. Retrieved from https://www.cancerresearchuk.org/health-professional/cancer-statistics/incidence/age#heading.

Ageing is also associated with gradual cognitive and structural changes in the brain. Research during the last 10 years has demonstrated that gut microbes are linked to the formation of the blood–brain barrier, myelination, neurogenesis, microglial maturation and behaviour. Studies using germ‐free mice have also demonstrated that gut microbes influence behaviour, anxiety and fear linking fear and anxiety to the microbiome (Bravo *et al*, 2011; Heijtz *et al*, 2011; Cryan & Dinan, 2012; Kundu *et al*, 2017). This field of research is currently subject to intensive investigations and some promising small studies, essentially all in animal models, have identified potential key players with an ability to reduce stress responses, influence anxiety and change behaviour, including social interactions (Sudo *et al*, 2004; Bercik *et al*, 2011; Bravo *et al*, 2011; Gareau *et al*, 2011; Heijtz *et al*, 2011; Ait‐Belgnaoui *et al*, 2012, 2014; Hsiao *et al*, 2013; Buffington *et al*, 2016). Recent microbiome profiling reports indicate that changes in microbiome composition have been shown to correlate with neurodegenerative diseases such as Alzheimer's (Chen *et al*, 2016) and Parkinson's (Jost, 1997; Pfeiffer & Quigley, 1999; Hardoff *et al*, 2001; Kelly *et al*, 2014; Fasano *et al*, 2015) (Fig 1).

However, more research is needed, especially in humans, before we can conclude whether human probiotic interventions will become a new treatment module in our attempt to support healthy ageing and attenuate accelerated ageing and onset of age‐related disease.

Assessing case studies of Parkinson's disease (Parkinson's, UK, 2018) and cancer (Cancer Research UK) in the UK, these diseases are strongly associated with age (Chu & Kordower, 2007), with a significant rise in the incidence of Parkinson's disease and cancer from the 60–64 age bracket until 75–79 years of age. These focused studies elucidate correlation between ageing and disease. In addition, the remaining surviving population of individuals over the age of 80 may have entirely bypassed the age‐related chronic diseases, either due to genetic reasons, the environment they have lived in or even a change in their bodies to support extreme ageing. The extremely old population, including centenarians who live beyond 100 years of age, has an even more distinct and unique microbial footprint as compared to the elderly (Biagi *et al*, 2010, 2016). The centenarians are populated with *Akkermansia, Christensenellaceae* and *Bifidobacterium* (Biagi *et al*, 2016). This variation is even greater than the difference in microbiome population observed between young adults and the elderly with an age gap of approximately 40 years (Biagi *et al*, 2016). While no direct causality has been shown, this raises the possibility of the existence of pro‐ageing microbiome that could facilitate a healthier population, which could go on to live a much longer life, allowing extreme ageing.

## Ageing is the gradual loss of organ function at a systemic level

Ageing has been described as an age‐dependent increase in failure rates of the organs in the human body. In most cases, this is due to a gradual loss of function in response to reduced ability to repair and support tissues with oxygen and nutrients. Importantly, the human body is a closed system and all organs are connected to one another. Perturbation in one organ often impacts other organs via inter‐organ crosstalk. Failure of the system occurs when there is a deviation of the homeostatic body function from the expected optimal function (Deshpande *et al*, 1986; Gavrilov & Gavrilova, 2001; Lai & Xie, 2006). It can thus be proposed that it is the body system's redundancy for the irreplaceable elements (the organs that work together) that is responsible for ageing (Deshpande *et al*, 1986; Gavrilov & Gavrilova, 2001; Lai & Xie, 2006). While ageing occurs in all organs, promising examples of holistic interventions (calorie restriction, drugs such as rapamycin, lifestyle alteration and supplements) have shown that ageing can be slowed down. Furthermore, the introduction of induced pluripotent stem (iPS) cell transplantation and whole‐organ transplantation implies that we can expect changes in the average lifetime of a human being.

Assuming that ageing occurs when the inter‐organ communication fails to maintain homeostasis and not just due to the failure in one step/process, we can envisage new ways to attenuate multiple degenerative pathways in the body. This allows for optimism in ageing and its management, where several mechanisms are targeted to slow down ageing. Recently, the microbiome and diet have been receiving much attention as a non‐invasive method of targeting ageing (Zhao & Shen, 2010).

Another feature of ageing is the gradual loss of barrier integrity, reduced capillary bed to exchange waste products with nutrients and oxygen, reduced cardiac output and respiration capacity. Collectively, this is often manifested by increasing low‐grade inflammation that negatively impacts organ function through leakiness across barriers of microbe‐related inflammatory molecules, including lipopolysaccharides. This barrier breach combined with impairment in metabolic homeostasis occurs across the body including, for example, skeletal muscle, liver, bone structures, adipocyte tissue, oral cavity, skin and blood–brain barrier integrity, thus transmitting partial immune activation of microglial cells and astrocytes in the brain (Bischoff *et al*, 2014; Galland, 2014). While the observation of low‐grade inflammation is well documented, we are far from understanding the precise underlying mechanisms and this represents another important area for future research of microbiome–host interactions among elderly. Yet, some examples exist; metabolites like D‐lactic acid and ammonia, produced by bacterial enzymes, can exert neurotoxic effects further affecting human behaviour (Galland, 2014) and changes to the microbiome have been reported to correlate with chronic fatigue syndrome and fibromyalgia (Galland, 2014).

Hence, further studies are warranted to unravel the underlying molecular mechanisms as well as to obtain a detailed metabolome map to superimpose the changes in microbiome composition and disease phenotype. Profiling the metabolome in conditions of neurodegenerative diseases via different mass spectrometry techniques, to identify endogenous molecules as well as dietary metabolites, unlocks a better understanding for dietary interventions that modify the microbiome to enhance health and cognitive function.

## Lessons from ageing intervention therapies in the 21^st^ century of precision medicine

Nearly 30 years ago, longevity genes were identified in non‐vertebrate organisms following attempts to identify genes that are associated with ageing. Although humans are at the top of the food chain and possess the highest cognitive ability, studying other organisms can provide us with deeper insights into pathways controlling ageing. The hydra, for example, propagates asexually as their stem cells do not fail to renew or regenerate and hence are non‐senescent (Schaible *et al*, 2011; Schaible & Sussman, 2013). In the search for longevity genes, the roles of APOE and FOXO3A were examined. FOXO was then identified as a key regulator of epithelial homeostasis and host–microbiome crosstalk. FOXO3A is a signature protein related to ageing and its downregulation results in slow down of population growth rate. Hence, it is known as a rate‐of‐ageing regulator (Schaible & Sussman, 2013).

Evidence from studies performed on yeast, fungi, nematodes, insects, rodents and humans have now shown that mutations in specific genes have the ability to reduce or extend lifespan as well as accelerate ageing (Rattan, 2005). Moreover, several of these longevity genes have conserved function in ageing across species, including mammals. Most prominent genes and biochemical pathways that have been identified with ageing are in the mTOR pathway, the insulin‐like growth factor/insulin pathway, sirtuins, (Henrique Mazucanti *et al*, 2015) kinases and kinase receptors, transcription factors, DNA helicases, telomerase, membrane glucosidases, GTP‐binding protein‐coupled receptors, cholesterol metabolism, heat shock protein genes and cell cycle arrest pathways to name a few (Rattan, 2005).

Targeting these pathways increase the lifespan of mice and delay the onset of disease (Henrique Mazucanti *et al*, 2015), thereby holding promise for future tailor‐made interventions for humans. Notwithstanding the discovery of longevity genes, ageing is an inevitable process for all living organisms and manifests as a gradual loss of function across the body due to accumulating damage across the different tissues (López‐Otín *et al*, 2013).

Aside from viewing ageing from a systemic approach, it could also be coupled with targeting specific proteins and genes that have been associated with ageing, thereby targeting ageing from both the macro‐ and microperspective.

Biogerontology, which studies the biological basis of ageing, seeks to prevent age‐related diseases and enhance the quality of life in old age by developing effective anti‐ageing strategies. Survival and longevity are strongly associated with maintenance and repair, i.e. repairing DNA damage and removal of reactive oxygen species (Rattan, 2005), often following inflammatory damage. The greater the damage accumulated in cells, the shorter the lifespan of an individual (Holliday, 2000).

However, anti‐ageing research has often been associated with pseudoscience and fraud, where overcoming ageing is promised through miraculous remedies, which have no scientific evidence. Anti‐ageing therapies promise to prevent ageing, diseases and hence death by prolonging lifespan, and most of them have been disappointing and unsuccessful (Rattan, 2005).

Literature on ageing usually focuses on targeting specific age‐related diseases to cure or prevent a particular disease. However, it does not target the process of ageing in itself. For example, cancer therapy aims to remove cancer cells and achieve pre‐disease state of the affected organ. While the therapy reduces the rate of mortality and aims to restore previous state of health, it does not address other diseases associated with ageing such as Parkinson's, Alzheimer's, dementia, frailty or cumulative organ failure. This is turn highlights the lack of exploration on “how to attenuate processes associated with accelerated ageing” as a principle in itself (Rattan, 2005). Other anti‐ageing strategies aim to slow down ageing by inhibiting or delaying physiological and functional deterioration. Supplementation with hormones such as growth hormones, melatonin and oestrogen, or supplements containing antioxidants have shown clinical benefits in treating aged patients. However, regulation of the ageing process itself has again, not been explored (Olshansky *et al*, 2002).

Targeted studies to eliminate one type of ageing‐related damage may assist in slowing down that specific ageing phenotype but the overall impact may not be significant since the other compounding factors of ageing remain unaltered. Since ageing is a systemic phenotype (Gavrilov & Gavrilova, 2001), holistic interventions targeting lifestyle (i.e. diet and exercise) are necessary if we want to slow the ageing process.

Results from a highly recognized Finnish Geriatric (FINGER) intervention study revealed the possible beneficial effects of attenuating cognitive decline through multi‐domain lifestyle interventions (Kivipelto *et al*, 2013; Rosenberg *et al*, 2018). The recommendation for Alzheimer's disease prevention would be a treatment cocktail including neuro‐transmitter modulators, neuroprotectors, dietary intervention/medical food, risk factor intervention, next‐generation targets, and amyloid and tau lowering molecules (Kivipelto *et al*, 2013). The one‐size‐does‐not‐fit‐all concept was reiterated along with tailored interventions for specific at‐risk profiles, combining non‐pharmacological and pharmacological treatments and using new technologies. It seems likely that background genetic variation will also influence the effectiveness of such personalized therapeutic regimes, particularly if they work via altering the interaction between the host microbiome and the immune system.

Little is known about the mechanisms and signalling pathways underlying gut–brain communication. The major hurdle is the complexity in which microbes can communicate with the brain, and moreover, the myriad of output signals from CNS that modulate and change the gut microbiome also referred to as “the evolving inner self”. (Kundu *et al*, 2017). There are a number of recent association studies where a dysbiotic microbiome combined with changes in diet appear to impact conditions like neurodegenerative diseases and chronic inflammation in the bowel, including irritable bowel syndrome. While encouraging, these data are largely obtained from transgenic animal models. There is an imminent need to obtain data from epidemiological and prospective studies including both healthy old people as well as those with defined diseases (Collado *et al*, 2016).

## Can ageing be altered?

While global initiatives and research focus on developing solutions for famine, diarrhoea and malnourishment, we have acquired an entire new set of chronic diseases that occur later in life, which has become one of the greatest challenges for human health. There is a rapid and explosive change in chronic disease spectrum (Rivera *et al*, 2002; Who & Consultation, 2003), which cannot be explained by genetics alone. Rather, environmental interactions including introduction of antibiotics, reduced exercise and reduced cost for unhealthy food are likely factors. No doubt, these changes have also massively altered our microbiome. There is therefore an intense global research effort to identify the biomarkers of ageing, predict age‐related diseases and intervene at an early stage (Zglinicki & Martin‐Ruiz, 2005; DelaRosa *et al*, 2006; Simm *et al*, 2008; Martin‐Ruiz *et al*, 2011). While successful interventions, be it drugs, change of diet or lifestyle, can postpone or attenuate the onset of ageing‐related diseases, most efforts are still focusing on these conditions in isolation, thereby missing the holistic approach to maintaining body function among the elderly. The transformation of human working conditions brought about by the industrial revolution drastically exposed the limitations of an ageing body with evident functional deficiencies and loss of productivity of the elderly in our communities (Lee, 2007). This transition in part destroyed the ancient view of elderly as the golden generation filled with wisdom and hence, an important asset to society.

Moving forward to better understand biological ageing and to reduce disease burden, a comprehensive perspective must be applied. In addition, improved quality of life is intimately coupled to breaking the pattern of disease occurring due to age, minimizing the years of morbidity in each elderly individual and introducing effective interventions that can reduce disability and dependency (Lunenfeld & Stratton, 2013). Successful ageing focuses on absence of disease, enabling good physical and cognitive function and active levels of social engagement (Bowling & Dieppe, 2005). Interestingly, a recent report added to this phenomenon of successful ageing by discussing the age‐related association between disease susceptibility and diversity of the microbiome (Cho & Blaser, 2012).

## Importance of community for healthy ageing

Ageing is about understanding the accumulation of changes over the years, including social, physical and psychological changes. While function and dynamics of organs slow down in the ageing body, concomitant with increased reaction time, age allows for the expansion of human wisdom and knowledge of the world. Likewise, the microbiome undergoes changes in composition and richness presumed to be compensatory to the reduced physiological dynamics in the heart, lung, and locomotion. However, wisdom combined with a microbiome adaptation to age as such does not remove the fact that the greatest risk to humans as it leads to an increasing risk of disease acquisition.

Marmosets have been used to illustrate the benefits of ageing and how they use social communication, which is acquired from birth. Approximately 20 different social calls are acquired by marmosets and they transfer their wisdom to subsequent generations via social communication and function under the operant paradigm in a social family context (dominant, competitive, sharing) (Clara *et al*, 2008). Social calls are different for different animals and the phenotype is adapted based on what is beneficial. These marmosets undergo a wasting syndrome when they are devoid of social communications due to the significant mental stress, which highlights the construction of a neural niche after birth that expands based on the environment (Clara *et al*, 2008). The ecological niche, cognitive niche and neural niche are constructed via the gut–brain axis, highlighting the role of the gut–brain axis leading to rapidly evolving brain function. The wisdom accumulated and generated in the older monkeys is passed on via communication (Clara *et al*, 2008) strikingly similar to humans.

The marmosets have been studied to understand parallels with the situations in communities that we live in. Like the marmosets, could the elderly in our communities be deteriorating because of the loss of communication and hence becoming more susceptible to diseases? Indeed, loss of social interactions and increasing disconnect from the society is generally assumed to be factors that accelerate ageing and increase susceptibility to disease (Sorkin *et al*, 2002; Holwerda *et al*, 2012). Notably, this applies also to the microbiome community with a concomitant reduction in diversity and richness among human beings becoming isolated (Claesson *et al*, 2012).

Further, lack of interpersonal functions is a distinct feature in schizophrenia. Many schizophrenic patients are devoid of social relationships and this social detachment is a significant obstacle to their recovery (Walker *et al*, 1993). Social interactions and communication have an impact on human physiology and also the microbiome.

Studies have shown that the elderly that live in a community‐centric environment had a unique microbial signature and a larger tendency to consume a fibre‐enriched diet, enhancing the microbial diversity and reducing inflammation by downregulating inflammatory factors including TNF‐α, C‐reactive protein and neopterin (Claesson *et al*, 2012). Moreover, this elderly population also displayed an increase in bacterial genes that are involved in metabolizing SCFAs such as acetate and butyrate (Claesson *et al*, 2012). Community dwelling elderly showed a higher population of *Firmicutes* in their microbiome as compared to a higher population of *Bacteroidetes* in elderly that lived in isolation (Claesson *et al*, 2012).

These studies elegantly illustrate the perception that ageing does not only need to be managed using physiological interventions. Current society is unfortunately rapidly moving away from social interactions to save money and time. Hence, expanding community development may be a useful tool since it plays a paramount role in healthy ageing.

Over the past decades, families no longer choose to live in large, extended families, but rather opt for the nuclear family system resulting in the “family decline” hypothesis (Bengtson, 2001). When coupled with the rise of social media, this widens the communication barrier between the elderly and the rest of the family (as well as the microbial exposure). The lack or inability of elders to communicate among the family, i.e. use their social calls or pass down their wisdom, could also be a factor to look into when designing holistic solutions to enable active ageing or delay wasting in the elderly populations.

## Research is about re‐discovery: learning from the ancient greek philosophers and their perceptions of ageing

The ancient philosophers addressed ageing from a societal and holistic perspective. While extensively discussing youth and old age, they reached a consensus that ageing is a matter of perception with no clear boundaries. Plato, for instance, addressed the concept of ageing from two stances, continuity and disengagement, where he defined continuity as the ability of the elderly to persist with the previous activities during their youth and referred to disengagement when people drifted away from previous goals, strategies and aims in life [105]. Applying Plato's wisdom to 21^st^ century precision medicine and the concepts of physiological ageing, healthy ageing can only be achieved if we maintain consistency of robust living as we did in our youth. Negligence of our health and wellness over the years results in the gradual slowing down and hence, disengagement, spiralling towards conditions predicted by Aristotle; illnesses and pain as companions of old age (Gendlin & Gendlin, 2012).

Galen of Pergamon further developed these concepts around humans in which he openly belittles the idea of combating or preventing ageing. He highlighted that just as how a good governor would supply the city with food for winter, it is man's duty to accumulate his biggest wealth, his health so that he can enjoy the fruits of it during his old age (Diamandopoulos, 2017). Galen's thoughtful views are applicable to our modern mindset, to understand ageing from a biological and evolutionary perspective with the intention of attenuating or postponing the natural process of ageing.

In the post‐Pasteur microbial century of “good microbe” centred perception, it follows that if we continue to support our microbes with the temporal and spatial cues (diet, exercise, community exposure and decreased stress) they require to maintain diversity and thus homeostasis, it will undoubtedly have positive repercussions on body function. This echoes the sentiments of Plutarch, who advocated that we have the power to build up physical and mental qualifications, which we are then able to exploit in our old age (Trench, 1874). If we live an active and healthy life during our youth, our old age will be more productive and manageable, similar to the viewpoint of Galen.

## What lies ahead?

The average lifespan in Classical Greece was reported to be 25 years but increased to 42 years after they crossed adulthood due to high infant mortality (Angel, 1947), and it eventually reached 72 years during the Roman period (Montagu, 1994). Current statistics show that human beings are living longer in the industrial world (Cervellati & Sunde, 2005) thus reflecting the fact we as a species have accumulated experience and know‐how for a longer lifespan. Clearly, we are at the beginning of unravelling mechanisms underlying ageing and it will take a long time to transition from a reductionist mindset to understanding ageing with a holistic perception. Yet, the recent re‐discovery of the indigenous microbes as a central player in human health and disease provides optimism. If we assess the human body function from a systems biology understanding of the ageing process, which ancient philosophers have empowered us with, then ageing will be as positive or negative as we believe. Using the gut microbiome as a target for future interventions with diet, microbial metabolites and possibly certain probiotics suggests that we may have a better control over how we choose to live our golden years, either in bliss or in pain. As Goethe wrote in his letter to Humboldt in 1732, 5 days before his death, “The ancients used to say: Animals are instructed by their organs. I will add: Men are too, but they have the advantage of being able to instruct their organs in their turn”. Perhaps our indigenous gut microbes are part of the second messenger instruction to our organs.

**Pending issues**

(i) The mechanisms underlying microbiome changes by age and the reasons are currently unknown. Current theories suggest impairment in barrier integrity, thus allowing microbes to breach barriers and indirectly elicit low‐grade inflammation. Another theory implies that gradual loss of organ function forces gut microbes to respond to the reduced physiological efficacy and as a consequence, increase in the generation of energy from the alimentary tract to the host through changes in microbiome composition. Both models are not mutually exclusive. More research is highly warranted.
(ii) Increasing the knowledge by which microbes and microbial metabolites influence signalling pathways and organ function within an ageing organism across gender. Of outmost importance is to identify microbial metabolites that directly or indirectly support healthy ageing or attenuate accelerated ageing. This includes also molecules that stabilize barrier integrity.
(iii) Undertake research to identify major “orchestral” microbe leaders that directly or indirectly influence microbiome diversity and richness relevant to its functions. An example is Akkermansia.
(iv) We have yet to explore the first tailor‐made microbiome intervention study in line with the biology of a given individual. The lessons from non‐responder/responder experiments using probiotic intervention approach from the Elinav laboratory (Zmora *et al*, 2018) tell us that us that more research is needed to establish proof of concept as well as who to target. Going forward this has huge implications for a broader and deeper understanding of healthy ageing.
(v) There is an unmet need to develop a better understanding of how gut microbes and their metabolites contribute to organ‐to‐organ communication either in early life or when organ function declines by age. This includes also the topological distribution of microbes in different compartments. For example, which are the temporal–spatial cues that guide homing to a specific part of the body?
(vi) Virtually nothing is known when microbes and the host end up having opposing needs. When does this occur, what are the signalling cues and underlying molecular mechanisms that trigger these situations? Is there room for man‐made interventions that can attenuate the conflict of interest between microbes and the host without massive violation of microbe–microbe interactions, like we do with pathogens and antibiotics? Future experiments involving antimicrobial peptides or phage therapy are therefore of interest.
(vii) Utilizing existing knowledge by providing SCFA to increase barrier integrity; does it come with unwanted side effects yet to be discovered especially since SCFA also has the ability to remodel chromatin structure? Systems biology analysis in human cohort studies is therefore highly warranted and this is an area where the food industry should together with academia, clarify the effects of microbial intervention and maintaining barrier function and overall metabolic homeostasis.
(viii) The rapidly growing number of old people worldwide is intimately associated with increased number of human beings with chronic lifestyle diseases because of increased lifespan. There is unmet need to undertake longitudinal studies among old people that is not subject to disease. Within these groups of people, there are those at risk to develop disease. A systems biology approach including non‐invasive fMRI analysis combined with multiple omic analysis, metagenomics and metabonomics of stool and fluid samples will open for discovery of novel biomarkers that can be used for future intervention studies prior to disease at an individual level.

**Box 1: Invited speakers who were referred to in this review**

(i) Dr. Paul Blatchford, Commonwealth Scientific & Industrial Research Organization (CSIRO) Health and Biosecurity, Adelaide, Australia
(ii) Professor Atsushi Iriki, Riken Brain Science Institute, Japan
(iii) Professor Dusko Ehrlich, King's College, London, UK
(iv) Professor Elaine Holmes, Imperial College, London, UK
(v) Professor Eran Elinav, Weizmann Institute of Science, Israel
(vi) Professor Miia Kivipelto, Karolinska Institute
(vii) Professor Thomas Bosch, Zoologisches Institut, University of Kiel, Germany

### Conflict of interest

The authors declare that they have no conflict of interest.
